# Distribution Pattern of Atherosclerotic Stenosis in Chinese Patients with Stroke: A Multicenter Registry Study

**DOI:** 10.14336/AD.2018.0602

**Published:** 2019-02-01

**Authors:** Yang Hua, Lingyun Jia, Yingqi Xing, Pinjing Hui, Xuan Meng, Delin Yu, Xiaofang Pan, Yalan Fang, Binbin Song, Chunxia Wu, Chunmei Zhang, Xiufang Sui, Youhe Jin, Jingfen Zhang, Jianwei Li, Ling Wang, Yuming Mu, Jingxin Zhong, Yuhong Zhu, Heng Zhang, Xiaoyu Cai

**Affiliations:** ^1^Department of Vascular Ultrasonography, Xuanwu Hospital, Capital Medical University, China.; ^2^Department of Neurology, First Hospital of Jilin University, China.; ^3^Department of Ultrasonography, The First Affiliated Hospital of Soochow University, China.; ^4^Department of Ultrasonography, Lanzhou University Second Hospital, China.; ^5^Department of Ultrasonography, Tianjin Huanhu Hospital, China.; ^6^Department of Ultrasonography, Dalian Municipal Central Hospital Affiliated of Dalian Medical University, China.; ^7^Department of Ultrasonography, The First Affiliated Hospital of Shanxi Medical University, China.; ^8^Department of Neurology, Luoyang Central Hospital Affiliated to Zhengzhou University, China.; ^9^Department of Ultrasonography, Liaocheng Brain Hospital, China.; ^10^Department of Ultrasonography, First Affiliated Hospital of Harbin Medical University, China.; ^11^Department of Ultrasonography, Anhui Provincial Hospital, China.; ^12^Department of Ultrasonography, First Hospital of China Medical University, China.; ^13^Department of Neurology, Baotou City Central Hospital, China.; ^14^Department of Ultrasonography, Fujian Provincial Hospital, China.; ^15^Department of Ultrasonography, Mianyang Central Hospital, China.; ^16^Department of Ultrasonography, First Affiliated Hospital of Xinjiang Medical University, China.; ^17^Department of Ultrasonography, Guangdong Provincial Hospital of Traditional Chinese Medicine, China.; ^18^Department of Neurology, The Second Affiliated Hospital of Kunming Medical University, China.; ^19^Department of Ultrasonography, Zhuhai People’s Hospital, China.; ^20^Department of Ultrasonography, Brain Hospital of Hunan Province, China.

**Keywords:** Ischemic stroke, Atherosclerosis, Distribution, Chinese, Risk factor, Vascular Ultrasound

## Abstract

The aim of this multicenter study was to demonstrate the distribution pattern of atherosclerotic stenosis and its trend with aging between extracranial and intracranial arteries and its distribution between the anterior and posterior circulations in Chinese patients hospitalized with ischemic stroke. In addition, the risk factors for the distribution pattern were illustrated. From June 2015 to May 2016, 9,346 patients with ischemic stroke from 20 hospitals were enrolled. Carotid artery ultrasonography and transcranial color-coded sonography/transcranial Doppler were used to evaluate the extracranial and intracranial arteries. The distribution pattern of atherosclerotic stenosis and its trend with aging were analyzed. Logistic regression was used to analyze the risk factors for the distribution pattern. Among the 9,346 patients, 2,882 patients (30.8%) had at least one artery with a degree of stenosis ≥50%. Among patients with arterial stenosis, the proportion of patients with intracranial artery stenosis was higher than those with extracranial artery stenosis (52.6% *vs.* 27.6%), and the proportion of anterior circulation artery stenosis was higher than that in the posterior circulation (52.2% *vs.*26.2%). With aging, the proportion of intracranial artery stenosis alone decreased; at the same time, the proportion of extracranial artery stenosis and extracranial plus intracranial artery stenosis increased (trend χ^2^=6.698, *P*=0.001). Hypertension (OR 1.416, *P*=0.008) and family history of stroke (OR 1.479, *P*=0.014) were risk factors for intracranial artery stenosis. Male, aging, and smoking were factors more related to extracranial artery stenosis. Aging (OR 1.022, *P*<0.001) and hypertension (OR 1.392, *P*=0.019) were related to posterior circulation artery stenosis. Intracranial arteries and anterior circulation arteries were susceptible to stenosis in Chinese patients with ischemic stroke. However, the distribution pattern of atherosclerotic stenosis was dynamic and varied with aging. Aging and different risk factors contribute to this distribution pattern.

The latest Global Burden of Disease 2015 Study (GBD 2015) reports that the prevalence of ischemic stroke increased by 15.8% between 2005 and 2015. In China, ischemic stroke is one of the leading causes of mortality [1,2]. Atherosclerotic stenosis of the extracranial and intracranial arteries is the main cause of ischemic stroke. It is known that intracranial atherosclerosis is more prevalent in Asian patients [3]. However, there is a lack of detailed large-scale studies on the distribution pattern of atherosclerosis between the extracranial and intracranial arteries as well as between the anterior and posterior circulation in the Chinese population, especially in patients with ischemic stroke. We hypothesized that the distribution pattern of atherosclerotic stenosis was different among different age stages, and the varied risk factors might contribute to this distribution pattern.

Noninvasive carotid artery color Doppler ultrasonography (CDU) and transcranial color-coded sonography (TCCS)/transcranial Doppler (TCD) have been widely used in China as screening methods for detecting extracranial and intracranial artery stenosis in patients with stroke [4]. In this cross-sectional, nationwide, multicenter registry study, using CDU and TCCS/TCD, confirmed with computed tomography angiography (CTA) and/or magnetic resonance angiography (MRA), we investigated the prevalence of atherosclerotic stenosis and its distribution pattern between extracranial and intracranial arteries and between the anterior and posterior circulation arteries in Chinese patients hospitalized with ischemic stroke. Furthermore, we analyzed the trends of distribution pattern with aging and the risk factors related to this distribution pattern.

## MATERIALS AND METHODS

### Subjects

This study was registered on and released by ClincalTrials.gov with the identification number NCT02397655. The study protocol was approved by the institutional ethics committee of Xuanwu Hospital, Capital Medical University (ethics approval number: 2015-008).

From June 2015 to May 2016, 9,346 hospitalized patients with ischemic stroke from 20 hospitals were continuously enrolled. The 20 hospitals involved in this study represented North, South, East China, Central China, Southwest, Northeast, and Northwest China. The diagnoses of ischemic stroke were made by a neurologist according to the American Heart Association/American Stroke Association (AHA/ASA) guidelines [5]. The inclusion criteria were as follows: (1) patients aged ≥40 years old and (2) patients was diagnosed with ischemic stroke within the prast 3 months, including acute and old cerebral infarction, with symptoms and needed to be treated in hospitals. The exclusion criteria were as follows: (1) artery stenosis or occlusion caused by nonatherosclerotic factors, such as dissection, arteritis, moyamoya disease, and muscle fiber dysplasia; (2) cardioembolic stroke; and (3) cerebral hemorrhage.

The risk factors collected in this study included hypertension, diabetes mellitus, dyslipidemia (defined as low-density lipoprotein cholesterol [LDL] ≥3.4 mmol/L, total cholesterol [TC] ≥5.2 mmol/L, or triglyceride [TG] ≥1.7 mmol/L at admission, a history of dyslipidemia or receiving lipid-lowering treatments, or diagnosed at discharge), and smoking (defined as a patient who had smoked continuously for 6 months ≥ 1 cigarette a day). In addition, family history of ischemic stroke (defined as the parents or siblings of the patient having a history of stroke) was recorded.

### CDU and TCCS/TCD examinations

All the CDU and TCCS/TCD examinations were performed by experienced ultrasound physicians (with more than 5 years of experience) and followd the vascular ultrasound protocol described by Zwiebel and Pellerito [6], and the vascular ultrasound examination guidelines published by the Chinese Medical Doctor Association of Ultrasonography [7]. The examinations used the Philips ultrasound systems (IU-22, Philips, Inc., Bothell, WA, USA) with a 3-9 MHz linear array probe and 1-5 MHz curvilinear array probe or Hitachi ultrasound systems (Ascendus, Hitachi, Inc., Tokyo, Japan) with 4-8 MHz microcurvilinear array probe to examine the extracranial arteries, including the common carotid artery (CCA) and the internal carotid artery (ICA), vertebral arteries (VA), and subclavian arteries (SAs). The sizes and echo characteristics of atherosclerotic plaques and the peak systolic velocity (PSV) and end-diastolic velocity (EDV) of the arteries were recorded. According to the ultrasound criteria [8-10], the degrees of stenosis of these arteries were categorized as <50%, 50%-69%, and 70%-99%, respectively, and occlusion based on the NACET arteries stenosis grading method.

Intracranial arteries, including the middle cerebral artery (MCA), the terminal of the ICA (TICA), anterior cerebral arteries (ACAs), posterior cerebral arteries (PCAs), the intracranial segment of the VA, and the basilar artery were examined by TCCS with a 1-5 MHz phased array probe or TCD (EMS-9A, Delicate Electronics Co., Ltd, Shenzhen, China) with a 1.6 MHz probe. The PSV and EDV of the arteries were recorded. According to the ultrasound criteria [11,12], and the degree of stenosis for MCA, TICA, VA, and basilar artery were categorized as mild, moderate, severe stenosis, and occlusion based on the WASID artery stenosis grading method. It should be noted that due to very low prevalence of ACA and PCA atherosclerosis alone [12], we did not analyze the stenosis frequencies of ACA and PCA into the final statistical analysis.

### Quality control protocols

To ensure the quality of the study, we have taken the following actions: (1) All the hospitals participated in this multicenter study were the base hospitals for stroke prevention and treatment awarded by the Stroke Screening Prevention Project Committee, National Health and Family Planning Commission of China, suggesting that these hospitals have a high standard of the techniques required for stroke prevention and treatment. (2) All the physicians performing vascular ultrasound have more than 5 years of experience in this field and received uniform training for CDU and TCCS/TCD before the study, with a diagnostic accuracy ≥ 90% (compared with CTA or MRA). (3) This study used the uniform ultrasound criteria for diagnosing artery stenosis [8-12]. All the patients with one of the above arteries having a degree of stenosis ≥50% diagnosed by ultrasound underwent CTA and/or MRA to confirm the degree of stenosis. Twenty-five patients had an in-tandem lesion of extracranial artery severe stenosis or occlusion combined with ipsilateral intracranial artery stenosis, in whom the degree of intracranial artery stenosis evaluated by ultrasound were not consistent with it by CTA or MRA (in such cases, it underestimated the degree of stenosis of intracranial arteries by ultrasound), we referred to the CTA/MRA results. (4) All the data were transferred into a central database every month. In addition, every month, the experienced physicians checked the data and returned any incomplete data to the relevant center and requested further data. All the data entered into the final analysis were complete and verified.

### Statistical Analysis

The Statistical Package for Social Sciences (SPSS version 22.0) software was used for the statistical analysis. Numerical values with normal distribution are shown as the mean ± SD, and *t* test was used to compare the difference between two groups. Numerical values with non-normal distribution are shown as the median (interquartile range), and a non-parametric test was used to compare the difference between two groups. The Pearson chi-square test was used to compare the enumeration values, and the chi-square trend test was used to investigate the linear trend of the enumeration values with aging. Logistic regression analysis was used to define the independent risk factors for the atherosclerosis distribution pattern. A *P* value <0.05 was considered statistically significant.

**Table 1:** The demographic and clinical characteristics of patient groups with or without stenosis.

Variable	Without Stenosis (N=6464)	Stenosis (N=2882)	t/χ^2^/Z	*P*
Age* (years)	64.6±10.9	64.3±10.8	1.232	0.218
Gender, number (%)			139.330	<0.001
Male	4106(63.5)	2188(75.9)		
Female	2358(36.5)	694(24.1)		
Comorbidities, (%)				
Hypertension	5437(84.1)	2509(87.1)	13.580	<0.001
Diabetes mellitus	2349(36.3)	1146(39.8)	9.984	0.002
Dyslipidemia	3194(49.4)	1536(53.3)	12.031	0.001
Smoking	2183(33.8)	1330(46.1)	130.158	<0.001
Family history(%)	474(7.3)	331(11.5)	43.658	<0.001
NIHSS+ at admission	2(0-4)	2(1-5)	11.835	<0.001
BMI*(Kg/m^2^)	24.3±3.18	24.6±3.26	4.179	<0.001
SBP* at admission (mmHg)	144.2±21.5	148.1±22.5	7.982	<0.001
DBP* at admission (mmHg)	85.1±13.1	85.4±13.2	1.020	0.308
TG*(mmol/L)	1.65±1.19	1.68±1.21	1.120	0.263
TC*(mmol/L)	4.38±1.11	4.36±1.21	0.782	0.434
LDL*(mmol/L)	2.68±0.93	2.75±1.02	3.260	0.001
HDL*(mmol/L)	1.19±0.40	1.15±0.44	4.327	<0.001
Fasting blood glucose*(mmol/L)	6.06±2.37	6.58±2.74	9.324	<0.001

Notes: BMI: body mass index; SBP: systolic blood pressure; DBP: diastolic blood pressure; TG: triglyceride; TC: total cholesterol; LDL: low-density lipoprotein; HDL: High-density lipoprotein.

* Continuous variables with normal distribution expressed as mean ± SD.

+ Continuous variables with non-normal distribution expressed as median (interquartile range). Other values expressed as n(%).

**Table 2:** The frequency and the degree of stenosis in different arteries.

Arteries	Normal or mild stenosis No. (%)	Moderate stenosis No. (%)	Severe stenosis No. (%)	Occlusion No. (%)
CCA	9263 (99.1)	51 (0.5)	16 (0.2)	16 (0.2)
ICA	8702 (93.1)	155 (1.7)	199 (2.1)	290 (3.1)
Extracranial VA	8682 (92.9)	184 (2.0)	160 (1.7)	320 (3.4)
SA	9144 (97.9)	133 (1.4)	47 (0.5)	22 (0.2)
MCA	7923 (84.8)	574 (6.1)	519 (5.6)	330 (3.5)
TICA	9240 (98.9)	13 (0.1)	28 (0.3)	65 (0.7)
Intracranial VA	8691 (93.0)	125 (1.3)	56 (0.6)	474 (5.1)
BA	9057 (96.9)	196 (2.1)	60 (0.6)	33 (0.4)

Notes: CCA: common carotid artery; ICA: internal carotid artery; VA: vertebral artery; SA: subclavian artery; MCA: middle cerebral artery; TICA: terminal of internal carotid artey; BA: basilar artery.

## RESULTS

### Prevalence rate of artery stenosis

Among the 9,346 patients, 2,882 patients (30.8%) had at least one artery with a degree of stenosis ≥50%. The demographic and clinical characteristics of the groups with and without stenosis are listed in Table 1. Compared with the group without stenosis, the stenosis group had higher NIHSS scores at admission, body mass index, proportion of males, hypertension, diabetes mellitus, dyslipidemia, smoking, and family history of stroke. In addition, the systolic blood pressure, serum LDL, and fasting glucose levels at admission in the stenosis group were significantly higher than those in the group without stenosis. The frequencies for each artery and the degree of stenosis are listed in Table 2. MCA had the highest frequencies of moderate and severe stenosis, and intracranial VA had the highest frequencies of occlusion.

In total, the prevalence rate of artery stenosis in male patients was 34.8%, which was notably higher than the rate in females (22.7%, OR=1.811, 95%CI 1.639-2.000, *P*<0.001). Moreover, we categorized the patients into five age groups, as follows: 40-49, 50-59, 60-69, 70-79, and ≥80 years. In all groups except for the 40-49-year group, the prevalence of artery stenosis in male patients was higher than in females (*P*<0.001, Table 2). In both male and female patients, with aging, the prevalence rates of artery stenosis were stable, without showing a significant rising trend (males: trend χ^2^=0.560, *P*=0.454; females: trend χ^2^=3.789, *P*=0.052, Table 3).

**Table 3:** The prevalence of ≥ 50% artery stenosis in patients with stroke of different age groups and different gender.

Age groups (years)	Total No. (%)	Male No. (%)	Female No. (%)	OR	95%CI	*P*
40-49	300 (30.6)	232 (31.1)	68 (29.2)	1.095	0.794-1.512	0.582
50-59	771 (32.0)	600 (34.7)	171 (25.1)	1.588	1.301-1.938	<0.001
60-69	972 (31.1)	762 (37.3)	210 (19.4)	2.471	2.074-2.944	<0.001
70-79	641 (29.7)	444 (33.0)	197 (24.1)	1.548	1.272-1.885	<0.001
≥80	198 (29.5)	150 (34.7)	48 (20.1)	2.117	1.457-3.074	<0.001
Total	2882 (30.8)	2188 (34.8)	694 (22.7)	1.811	1.639-2.000	<0.001
trend χ^2^	1.948	0.560	3.789			
*P*	0.163	0.454	0.052			

Note: OR: male vs. female

### Distribution characteristics of artery stenosis

In 2,882 patients with artery stenosis, the proportion of patients with intracranial artery stenosis was notably higher than those with extracranial artery stenosis (52.6% *vs.*27.6%), and the proportion of stenosis in the anterior circulation arteries was higher than that in the posterior circulation (52.2% *vs.* 26.2%). With respect to the gender difference, male patients had a higher proportion of extracranial artery stenosis, while females had a higher proportion of intracranial artery stenosis (χ^2^=27.090, *P*<0.001). There was no difference in the distribution pattern of anterior and posterior circulation artery stenosis between male and female subjects (χ^2^=4.850, *P*=0.088) (Table 4).

**Figure 1.**
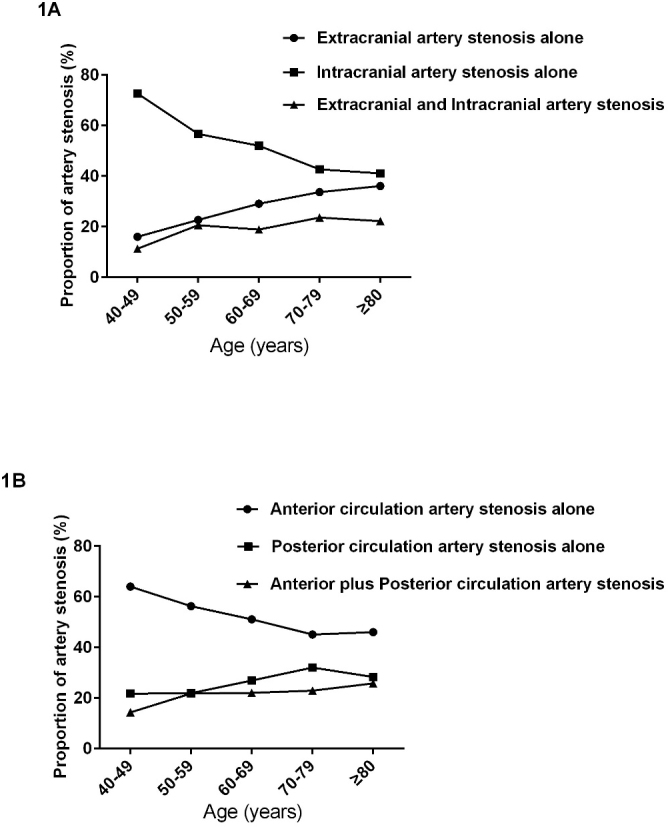
The distribution pattern trends of artery stenosis with aging between extracranial and intracranial artery (A) and between anterior and posterior circulation artery (B) in patients with ischemic stroke.

As shown in Figure 1A and 1B, the proportion of intracranial artery stenosis alone decreased with aging; at the same time, the proportion of extracranial artery stenosis and extracranial plus intracranial artery stenosis was increased with aging (trend χ^2^=6.698, *P*=0.001). Similarly, the percentage of anterior artery stenosis alone declined and the percentage of posterior circulation artery stenosis and anterior plus posterior circulation artery stenosis increased with aging (trend χ^2^=26.553, *P*<0.001).

### Risk factors affecting artery stenosis distribution

Finally, we compared the risk factors between patients with extracranial artery stenosis alone and those with intracranial artery stenosis alone. Logistic regression analysis revealed that hypertension (OR 1.416, 95%CI 1.096-1.829, *P*=0.008) and family history of stroke (OR 1.479, 95% CI 1.084-2.018 *P*=0.014) were risk factors for intracranial artery stenosis. Male, aging, and smoking were more closely associated with stenosis of the extracranial arteries (Table 5). Moreover, aging (OR 1.022, 95% CI 1.013-1.030, *P*<0.001), and hypertension (OR 1.392, 95% CI 1.055-1.836, *P*=0.019) were more related to stenosis in the posterior circulation arteries (Table 6).

## DISCUSSION

In this large-scale (9, 346 patients), cross-sectional, and multicenter registry study, we investigated the prevalence rate of large artery atherosclerotic stenosis and demonstrated its distribution pattern characteristics in Chinese patients hospitalized with ischemic stroke. The present study included almost all the arteries susceptible to atherosclerotic stenosis when demonstrating the distribution pattern. In addition, we demonstrated the trends of distribution pattern with aging and analyzed the risk factors contributing to this distribution pattern. Prevalence of stroke has been reported to be more common among men. [13] We also found that the prevalence of artery stenosis in males was higher than that in females, especially in patients age ≥50 years old.

**Table 4:** The distribution pattern of artery stenosis in patients with stroke.

Stenosis Distribution	Total No. (%) (N=2882)	Male No. (%) (N=2188)	Female No. (%) (N=694)	χ^2^	*P*
Extracranial arteries alone	794(27.6)	648(29.6)	146(21.0)		
Intracranial arteries alone	1516(52.6)	1094(50.0)	422(60.8)	27.090	<0.001
Extracranial and intracranial arteries	572(19.8)	446(20.4)	126(18.2)		
Anterior circulation alone	1503(52.2)	1122(51.3)	381(54.9)		
Posterior circulation alone	756(26.2)	573(26.2)	183(26.4)	4.850	0.088
Anterior and posterior circulation	623(21.6)	493(22.5)	130(18.7)		

Significantly more patients had intracranial artery stenosis than extracranial artery stenosis, with an approximate ratio of 5:3. These findings corroborate with the previous notion that intracranial atherosclerosis is more prevalent in Asian patients [3] and are consistent with the CICAS study [14]. Although the patients enrolled in both studies were Chinese patients with ischemic stroke, there are some differences between the present study and the CICAS study: 1) The CICAS study focused mainly on intracranial artery stenosis and did not include the extracranial VA, intracranial VA, and SA when it analyzed the distribution patterns between extracranial and intracranial artery stenosis [14,15]. In this study, MCA had the highest frequencies of moderate and severe stenosis, which is consistent with the CICAS study [15]. In addition, we found intracranial VA had the highest frequencies of occlusion. The extracranial VA and intracranial VA had relatively high frequencies of stenosis (including occlusion), which is the main reason for posterior circulation ischemia; thus, it is necessary to include the VA data when demonstrating the distribution pattern of atherosclerotic arteries stenosis. 2) The ages of the enrolled patients were different; the CICAS study included a group of young patients (aged 18-39 years), and our study only enrolled patients ≥40 years old (patients ≥40 years old were enrolled into the Stroke Screening Prevention Project, National Health and Family Planning Commission of China). The geographical location might affect the atherosclerotic stenosis distribution because the latest published study showed that, even in China, the prevalence and mortality of stroke vary among different regions [14,16]. The 20 hospitals involved in this study covered North, South, China, China, Southwest, Northeast, and Northwest China; thus, the patients represented a large part of the country.

**Table 5:** Risk factors for the distribution pattern between extracranial and intracranial arteries stenosis by logistic regression analysis.

Factors	Extracranial artery stenosis No. (%) N=794	Intracranial artery stenosis No. (%) N=1516	OR*	95%CI	*P*
Aging	66.6±10.2	62.7±11.0	0.962	0.954-0.970	<0.001
Male	648(81.6)	1094(72.2)	0.663	0.526-0.836	0.001
Hypertension	665(83.8)	1333(87.9)	1.416	1.096-1.829	0.008
Diabetes mellitus	277(34.9)	627(41.4)	1.196	0.991-1.443	0.062
Dyslipidemia	416(52.4)	809(53.4)	0.962	0.805-1.149	0.668
Smoking	408(51.4)	639(42.2)	0.672	0.551-0.819	<0.001
Family history	63(7.9)	182(12.0)	1.479	1.084-2.018	0.014

Notes:

* OR (odd ratio): intracranial artery stenosis *vs.* extracranial artery stenosis

The differences among the traditional risk factors are believed to affect the atherosclerotic stenosis distribution [3]. The present study has demonstrated that hypertension was a risk factor for intracranial artery stenosis, which was consistent with the results of a study by Bae et al. [17] who studied asymptomatic patients with intracranial cerebral atherosclerosis. A recently published single-center study [18] with Chinese patients also found that hypertension was closely associated with intracranial artery stenosis. Because the structure of the intracranial artery reveals thinner media and adventitia and contains fewer elastic media fibers compared to the extracranial artery, intracranial artery is more vulnerable to hemodynamic stress in the presence of hypertension [19].

**Table 6:** Risk factors for the distribution pattern of anterior and posterior circulation arteries stenosis by logistic regression analysis.

Factors	Anterior circulation artery stenosis No.(%)(N=1503)	Posterior circulation artery stenosis No.(%) (N=756)	OR*	95%CI	*P*
Aging	63.2±11.0	65.7±10.6	1.022	1.013-1.030	<0.001
Male	1122(74.7)	573(75.8)	1.067	0.855-1.330	0.567
Hypertension	1280(85.2)	675(89.3)	1.392	1.055-1.836	0.019
Diabetes mellitus	584(38.9)	297(39.3)	0.996	0.827-1.200	0.967
Dyslipidemia	789(52.5)	419(55.4)	1.137	0.952-1.359	0.156
Smoking	657(43.7)	334(44.2)	1.149	0.861-1.535	0.346
Family history	153(10.2)	82(10.8)	1.119	0.917-1.367	0.268

Notes:

* OR (odd ratio): posterior circulation artery stenosis *vs.* anterior circulation artery stenosis

Furthermore, this study showed that the proportion of stenosis in the anterior circulation arteries was higher than that in the posterior circulation arteries in the Chinese population. Aging and hypertension were more related to posterior circulation artery stenosis. In studies in Korea [20], Japan [21], and a single-center study in China mentioned above [18], the authors also found that hypertension was a risk factor for the development of posterior circulation artery stenosis. However, in a registry study [22] in Canadian patients with stroke, the authors showed that hypertension was not associated with posterior circulation infarcts. This discrepancy in results may be rooted from the different types of hypertension among ethnic groups, with high-volume hypertension being more prevalent in Asians and resistant hypertension being more common in Caucasians [3].

With regard to the gender difference of the distribution pattern, the present study showed that female patients had a higher proportion of intracranial artery stenosis than males (60.8% *vs.*50.0%), which is consistent with the CICAS study (51.51% *vs.* 45.40% in patients aged > 63 years, *P*=0.028) [14]. Moreover, logistic regression analysis showed that gender was an independent factor for the distribution pattern between intracranial and extracranial arteries stenosis, which was in agreement with the meta-analysis reported by Ding et al. regarding the extracranial and intracranial artery stenosis distribution in the Asian population [23]. Although the percentage of smokers is higher among men, this study found that smoking was an independent risk factor for extracranial artery stenosis (intracranial *vs.* extracranial OR 0.672, *P*<0.001), which was consistent with the meta-analysis results (intracranial *vs.* extracranial pooled OR 0.71, *P*<0.001) [23].

The novelest and important discovery in this study is that we demonstrated the atherosclerotic artery stenosis distribution pattern trends with aging. Although the total frequencies of artery stenosis with ≥ 50% were not increasing with age, the distribution pattern of atherosclerotic stenosis between extracranial and intracranial arteries and its distribution between the anterior and posterior circulations showed an obvious trend along aging. With aging, the proportion of extracranial artery stenosis was gradually closer to that of intracranial artery stenosis. In this study, the ratio of frequencies of extracranial to intracranial arteries stenosis from 1:3 in patients 40-49 years old to near 1:1 in patients age ≥ 80 years old. Moreover, logistic analysis showed that aging was an independent risk factor for the distribution pattern. This new finding suggested that aging is a key factor that should be considered when demonstrating the distribution pattern of atherosclerotic artery stenosis. These distribution pattern trends might be attributed, at least partially, to the age-specific risk factor profiles and age associated accumulation of the number of risk factors. Genetic factors are believed to contribute to the development of intracranial artery stenosis [3]. We also found that family history of stroke was an independent risk factor for intracranial artery stenosis. The proportion of patients with family history diminished with aging [24], which is linked to the decreased proportion of intracranial artery stenosis alone. In addition, Hauer et al. [24] demonstrated that the traditional risk factors gradually accumulated with aging. Therefore, under the effects of accumulating risk factors, the proportion of combined artery stenosis either with extracranial and intracranial artery stenosis as well as with anterior and posterior circulation artery stenosis increased.

The limitations of the study are that all the patients enrolled in the study had obvious symptoms and needed to be treated in hospital. Some patients with ischemic stroke but without symptoms or patients with mild symptoms that could be treated in an outpatient clinic were not enrolled. Thus, the results may not completely represent all patients with stroke in China. In addition, the correlation between this stenotic lesion with the location of cerebral infarction needs to be further studied.

In conclusion, intracranial arteries and anterior circulation arteries were susceptible to stenosis in Chinese patients with ischemic stroke. The distribution pattern of atherosclerotic stenosis was dynamic and varied with aging. Aging and different risk factors contribute to this distribution pattern. Specifically, hypertension and family history of stroke were more related to intracranial artery stenosis. Male, aging, and smoking were factors more related to extracranial artery stenosis. Aging and hypertension were risk factors for posterior circulation artery stenosis.
